# Applying high-resolution melting (HRM) technology to identify five commonly used *Artemisia* species

**DOI:** 10.1038/srep34133

**Published:** 2016-10-04

**Authors:** Ming Song, Jingjian Li, Chao Xiong, Hexia Liu, Junsong Liang

**Affiliations:** 1 School of Chemical Engineering, Wuhan University of Technology, Wuhan 430070, China; 2 College of Forestry and Landscape Architecture, South China Agricultural University, Guangzhou 510642, China; 3 College of Pharmacy, Hubei University of Chinese Medicine, Wuhan 430065, China; 4 Guangxi Institute of Botany, The Chinese Academy of Sciences, Guilin 541006, China; 5 College of Life Science & Technology, Yulin Normal University, Yulin 537000, China; 6 Cultivation Base for Key Laboratory of Conservation and Utilization of Rare and Economic Species at Southeast Guangxi, Yulin Normal University, Yulin 537000, China

## Abstract

Many members of the genus *Artemisia* are important for medicinal purposes with multiple pharmacological properties. Often, these herbal plants sold on the markets are in processed forms so it is difficult to authenticate. Routine testing and identification of these herbal materials should be performed to ensure that the raw materials used in pharmaceutical products are suitable for their intended use. In this study, five commonly used *Artemisia* species included *Artemisia argyi, Artemisia annua, Artemisia lavandulaefolia, Artemisia indica*, and *Artemisia atrovirens* were analyzed using high resolution melting (HRM) analysis based on the internal transcribed spacer 2 (ITS2) sequences. The melting profiles of the ITS2 amplicons of the five closely related herbal species are clearly separated so that they can be differentiated by HRM method. The method was further applied to authenticate commercial products in powdered. HRM curves of all the commercial samples tested are similar to the botanical species as labeled. These congeneric medicinal products were also clearly separated using the neighbor-joining (NJ) tree. Therefore, HRM method could provide an efficient and reliable authentication system to distinguish these commonly used *Artemisia* herbal products on the markets and offer a technical reference for medicines quality control in the drug supply chain.

*Artemisia* (Asteraceae) is a large genus including approximately 300 species, from which, many members have been described to possess medicinal properties^1^. According to Chinese Pharmacopoeia, the herbal materials of *Artemisia* divided into different phytomedicine categories base on their use and efficacy^2^. For example, *A. annua* and its derivatives are effective in treating malaria^3^. *A. argyi* has immuno-modulatory properties with its ability to treat eczema, inflammation, hemostasis, menstruation-related symptoms, and tuberculosis^4^. *A. indica* is frequently used in folk medicine for its antipyretic efficacy^5^. In China, several species of this genus like *A. argyi, A. annua, A. indica*, etc. are considered to be well known and economically important herbal materials due to their widespread use as household medicinal plants^6^. With the increasing use of herbal plants, the correct identification is therefore essential for their safe use. However, the extraordinarily similar morphological traits in the genus *Artemisia* often makes identification at the species level difficult. Given the valuable members of *Artemisia* described above, an easy and accurate method of authenticating the *Artemisia* species is indispensable for ensuring the drug quality of traded herbs.

So far, different analytical methodologies have been proposed for quality control purposes and authentication of herbal plants^7^,^8^,^9^,^10^. Among them, DNA barcoding is a new powerful tool which offers a practical solution either as stand-alone method for authentication or to reinforce these more traditional ones. DNA barcoding is not restricted by morphological characteristics and physiological conditions and allows species authentication without specialist taxonomic knowledge. The method is based on a short, standardized fragment of the genome called “DNA barcode”^11^. This short sequence of nucleotides could be from an appropriate part of the chloroplast, mitochondrial or nuclear genome and is used to identify organisms at the species level^12^,^13^. This addresses the difficulties involved in classifying herbal materials and promises to fuel a taxonomic renaissance in herbal identification^14^. Based on systematic research, researchers have proposed the internal transcribed spacer 2 (ITS2) as the core DNA barcode, for medicinal plant authentication^15^. Discriminatory capabilities of ITS2 sequence has been validated by many previous studies^16^,^17^,^18^. However, DNA barcoding in herbal medicines does have its own disadvantage. It is relatively time-consuming and expensive post-PCR procedures such as DNA sequencing which it is not a cost-effective method for developing countries. Very recently, several literatures report a new technique namely high resolution melting (HRM) in herbal medicine identification, and these studies showed a potential of HRM combined DNA barcode markers effectively distinguish medicinal plants, as well as to detect limit of adulteration in commercial herbal products^19^,^20^,^21^,^22^,^23^,^24^,^25^,^26^. HRM technology characterizes nucleic acid samples based on their disassociation behavior, using direct melting to detect small sequence differences in PCR-amplified sequences. These differences are detected through the use of DNA-specific dyes, high-end instrumentation and sophisticated analysis software. Samples are discriminated according to their composition, length, guanine-cytosine (GC) content, and strand complementarity^27^. An important advantage of HRM is that the analysis is performed immediately after the amplification. Thus, it is particularly suitable for medium to high throughput amplification.

In this study, we use the ITS2 barcode combined with HRM technology to identify five commonly used *Artemisia* species and authenticate their commercial products sold on the market in China. Distinguishing between these medicinal materials will enhance the quality control and management strategies for herbal products in drug supply chain.

## Results

### Identification of medicinal plants using HRM assay

**Figure 1:**
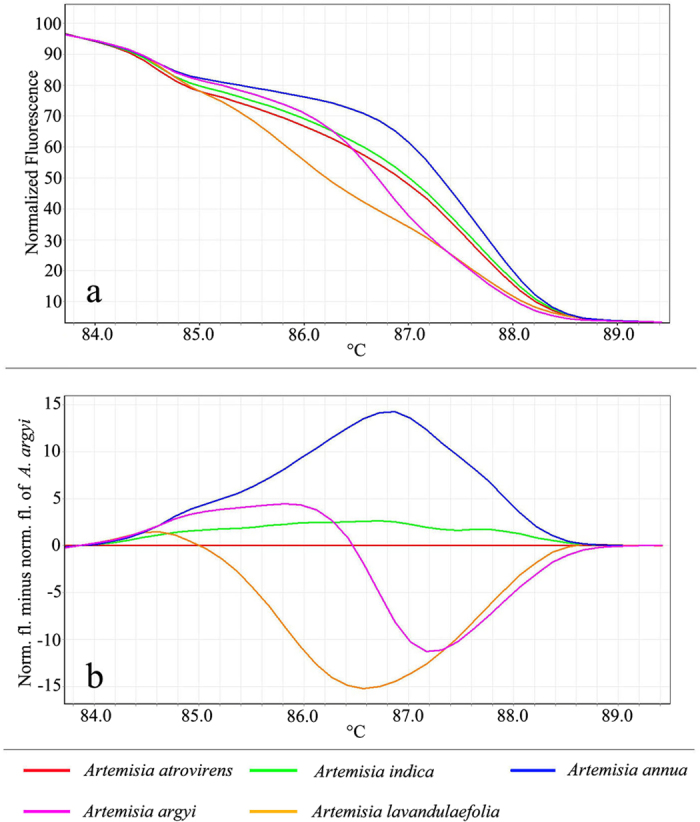
High resolution melting analysis of five species tested using the universal ITS2 marker. (**a**) Representative profiles of the melting curves of five *Artemisia* species. (**b**) Melting curves of the ITS2 amplicons from the five species using *A. atrovirens* as reference.

A real-time HRM-PCR protocol was applied for the identification of *Artemisia* species. The reproducible individual melting curves were obtained from different species with triplicate. Analysis of the normalized HRM curves with the barcode marker ITS2 were obtained based on the pattern of temperature-shifted curves and difference plot (Fig. 1). As shown in Fig. 1a, the barcode marker ITS2 was specific to these different herbal species with different melting profiles, making them easily distinguishable from each other. For example, *A. argyi*, and *A. annua* and *A. lavandulaefolia* are easily distinguished based on their unique melting curves. In contrast, *A. atrovirens* and *A. indica* presented similar curve profiles, and therefore could not be visually differentiated. In order to better visualize small differences between individual melting curves, HRM software applications allow calculation of a difference plot. Improved visualization and separation of variant melting curves from each species were shown in Fig. 1b. Assigning species *A. atrovirens* as a genotype with its melting curve as the baseline we were able by subtracting the area (difference graph) from the rest of the produced melting curves by the other species, to estimate the confidence value of similarity between the five species used. Furthermore, genotype confidence percentage (GCP) values were calculated, and a cut-off value of 90% was used to assign a genotype for each barcode region (Fig. 1b). With this approach, all samples were successfully genotyped, and these five species were sufficiently and confidently identified. These results demonstrated that the HRM analysis combined with ITS2 primer pairs is a powerful tool in the identification of closely related *Artemisia* species.

### Detection of commercial *Artemisia* herbal products

**Figure 2:**
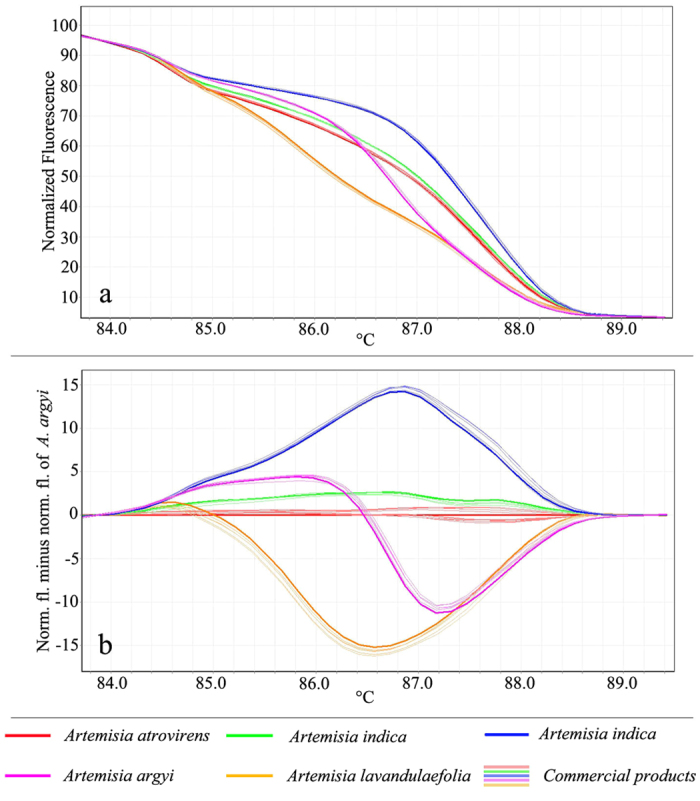
High resolution melting analysis using the universal ITS2 primers for commercial products. (**a**) Normalized curves of the five species and their commercial products. (**b**) Difference plot curves of the ITS2 amplicons from the five species and 10 commercial products using *A. atrovirens* as reference genotype.

After the confirmation that the five tested species can be identified by HRM analysis we applied the same approach to identify their commercial herbal products. Genomic DNA was extracted from the 58 commercial products. Sixty-one percent of the medicinal material samples contained DNA concentrations greater than 100 ng/μL, 27% contained between 50 and 100 ng/μL, and only 12% were lower than 50 ng/ μL. All of the commercial material samples were suitable to perform HRM-PCR amplification. The tested samples produced a unique melting plot that was easily to spot (Fig. 2). The normalized HRM curves for the amplicons based on barcode marker ITS2 are shown in Fig. 2a. All samples contained the botanical origin species that was promised or labeled as can be seen from their normalized HRM curves. Using the shape of the melting curves, we could assign the differences between curves of the different species under investigation and reveal that most of the species could be easily distinguished visually. Furthermore, closer examination of the HRM difference curve with *A. atrovirens* curve as the baseline suggesting that all the examined samples are similar to their labeled botanical species (Fig. 2b).

### Validation of HRM results using DNA sequencing

**Figure 3:**
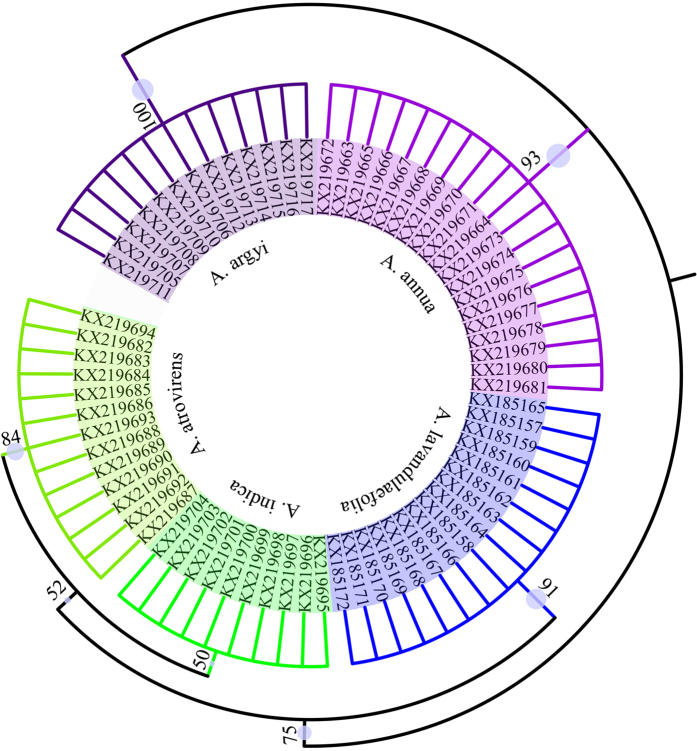
Phylogenetic tree of the five *Artemisia* species and their commercial products constructed with the ITS2 sequences using NJ method. The bootstrap scores (1000 replicates) are shown (≥50%) for each branch.

To validate the detection results of commercial *Artemisia* products, the HRM-PCR products were sequenced and high-quality bidirectional sequences were obtained. All the ITS2 sequences were submitted to DNA barcoding system for species identification (http://www.tcmbarcode.cn/en/). Finally, a phylogenetic tree based on NJ cluster algorithm was constructed using MEGA5 software (Fig. 3). The blast results and NJ tree all agreed with those obtained by HRM. These results also demonstrated that the use of the ITS2 sequence as a core DNA barcode for herbal medicine identification can clearly distinguish these five *Artemisia* species; therefore, all of the medicinal material sequences can also be incorporated into the *Artemisia* dietary supplement species DNA barcode database.

## Discussion

### Herbal medicine quality and safety

**Figure 4:**
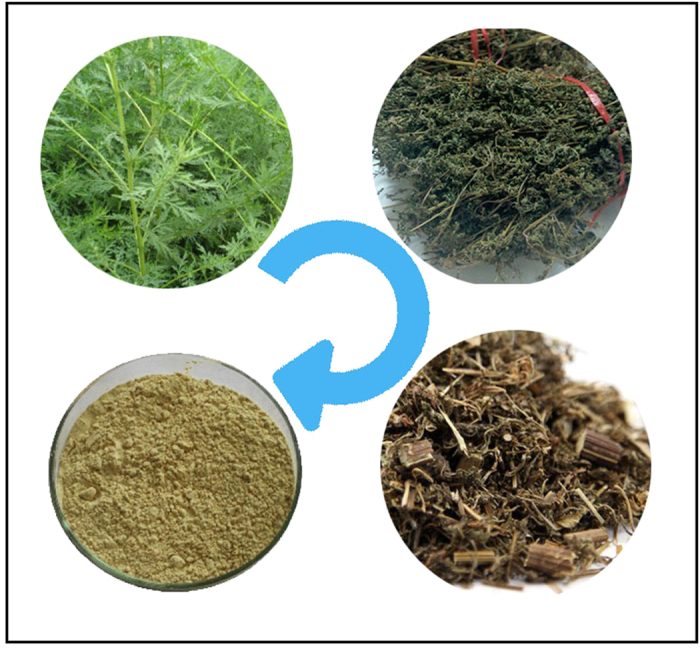
Herbal products sold in processed forms. From original plant to powder form.

During the past decade, advances in pharmaceutical drugs have improved health outcomes and the overall performance of health care system. However, in most of the developing countries, a large number of people still depend on traditional herbal remedies. Interesting, Global Industry Analysts Inc. reported that the sale of herbal medicine products has increased each year since 2004 in the global^28^. Parallel with increasing interest in herbal medicine for the prevention and treatment of various illnesses, there is also increasing concern about the quality and safety of medicinal plants. In China, a country where traditional therapies and products are widely used, there were 9,854 known reported cases of adverse drug reactions in 2002 alone, up from 4,000 between 1990 and 1999^29^. In some of these cases, the cause of the safety-related issues was the inaccurate identification of herbal materials^30^,^31^. Traditionally, biological species are authenticated according to their morphological features which are still the main basis of taxonomy. However, for herbal products, the authentication becomes complicated because the original identifying characteristics are absent as these products are primarily sold in processed or modified forms such as dried herbs, powders, tablets, and teas (Fig. 4). Take *Artemisia* plants for instance, this genus has similar macro-structural morphology among species, several species even sharing similar vernacular names in local languages. Conventional identification methods cannot solve all species determination problems in *Artemisia*, such as distinguishing between *A. atrovirens* and *A. indica* based on morphology^6^. Confusion in *Artemisia* species had been seriously impeded resource collection and clinic application. Moreover, these herbal materials are commonly sold in tablets or powders form in the market, making it more difficult to accurately identify the constituent plant species. The correct testing or identifying of raw herbal materials should be performed to ensure that the raw materials used in pharmaceutical products are suitable for their intended use.

### DNA barcode combined HRM method for herbal medicine identification: a potential application

In 2010, Chen *et al*. compared seven candidate DNA barcodes (*psb*A-*trn*H, *mat*K, *rbc*L, *rpo*C1, *ycf*5, ITS2, and ITS) from medicinal plant species and proposed that ITS2 can be potentially used as a standard DNA barcode to identify medicinal plants. Since then, several researchers have already demonstrated the potential of using ITS2 for taxonomic classification and phylogenetic reconstruction at both the genus and species levels for eukaryotes, including animals, plants, and fungi^32^. Recently, several studies have reported the use of HRM to evaluate herbal medicine substitution among defined groups of plant species, such as seven *Sideritis* species^19^, three medicinal species of Acanthaceae^22^, *Thunbergia* species^23^ and two *Hypericum* species^25^. These studies all showed that HRM analysis coupled with DNA barcode has a great potential to be applied for species identification. To evaluate the ability of HRM method to authenticate *Artemisia* species, we selected the ITS2 loci as the target. Similar melting curves were achieved from the same species regardless of the type of the DNA template (original fresh plant or processed products). Differences in the melting curves at the ITS2 region allowed discriminating the five commonly used herbal species analyzed in the study. *A. argyi*, and *A. annua* and *A. lavandulaefolia* are easily distinguished due to the larger variation between species (interspecific). For two closely related species (*A. atrovirens* and *A. indica*) the melting curves were very similar due to the low interspecific divergence, these species could, however, be distinguished with the help of a difference plot. Misidentification has not been an issue for these commonly used *Artemisia* species according to the HRM results in this study, but several commercial medicinal plants in China are currently facing the issue^20^,^33^. Very recently, several studies reported that HRM analysis allows assessing the amount of adulterant in a sample. In their study on the detection of *Thunbergia laurifolia* adulteration, for example, the authors declared that the HRM analysis method allows the detection of 1% *Crotalaria spectabilis* in *T. laurifolia*^23^. The same limit of detection was reported for the detection of *P. urinaria* species in *P. amarus* admixtures^24^, the detection of vetch in lentils^34^, the detection of “Fava Santorinis” in mixtures with *Lathyrus cicera*^35^. However, in our study, the standard curve can not be established a linear relationship, regardless of which two species were chosen. In our opinion, HRM analysis has to be evaluated in more detail in order to demonstrate its applicability for the accurate quantification of adulterants. Despite this, HRM has showed its potential power as a novel alternative molecular technology for accurate species identification, monitoring, and quality control of herbal materials.

In conclusion, given the increasing popularity and global demand for natural remedies and medicines in recent years, the ability to ensure a safe and sustainable supply of a quality product is primary. In this study, ITS2 was examined for its stability and accuracy in identifying Chinese materia medica in the case study with five commonly used *Artemisia* species by using HRM assay. The results indicate that ITS2 can be used as an effective biomarker for HRM to identify *Artemisia* species. ITS2 revealed the diversity and phylogenetic relationship among the species we tested, and can be used reliably to determine species identity, particularly in the absence of other defining traits. These findings will help for authentication of *Artemisia* species in herbal infusions as well as provide a new technique to ensure clinical quality and safety in using traditional Chinese medicines.

## Methods

### Plant materials and DNA isolation

In this study, 71 authenticated samples were used to develop HRM assay (Table1). These samples include 13 *Artemisia* specimen samples collected from different habitats (5 species: *A. argyi, A. annua, A. indica, A. lavandulaefolia*, and *A. atrovirens*) and 58 commercial products that are readily available to consumers and patients gathered from drug stores in different provinces in China. All of the corresponding voucher samples were deposited in the Herbarium of Guangxi Institute of Botany, The China Academy of Sciences. Genomic DNA was isolated from 30–40 mg dried material using the Plant Genomic DNA Kit (Tiangen Biotech Co., China), according to the DNA extraction protocol, and the DNA concentration was quantified using a spectrophotometer (Qubit 3.0, Invitrogen Co., USA). The DNA samples were then adjusted to 50 ng/μL working concentration and stored at 4 °C until further use.

### HRM-PCR amplification and data analysis

The HRM-PCR reaction mixture (25 μL) contained 50 ng of genomic DNA, 12.5 μL of 2 × HRM PCR master mix, 1 μL of 10 μmol/L of forward and reverse primers (ITS2F: 5′-ATG CGA TAC TTG GTG TGA AT-3, ITS3R: 5′-GAC GCT TCT CCA GAC TAC AAT-3′), and distilled water up to the final volume^36^. DNA amplification was performed in a Rotor-Gene Q MDx (QIAGEN) real-time PCR under the following conditions: 94 °C for 3 min, followed by 40 cycles of 94 °C for 30 s, 56 °C for 30 s and 72 °C for 45 s. The fluorescent data were acquired at the end of each extension step during the PCR cycles. HRM analysis was performed immediately following the completion of PCR amplification. The temperature was raised from 70 °C to 90 °C at 0.15 °C degree increments with a 2 s hold time for each acquisition step. Fluorescence was continuously monitored during the slow warming up of the gradient curve with a sharp fluorescence drop near the denaturation temperature. The Rotor-Gene Q software was used to analyze the melting profiles. The negative derivative of the fluorescence (*F*) over temperature (*T*) (d*F*/d*T*) curve displays *Tm*, and the normalized raw curve depicts the decreasing fluorescence against increasing temperature. Genotypes of test samples were identified by selecting a representative sample for each species. Based on a confidence threshold of 90%, a confidence percentage for each genotype was calculated.

### DNA sequence analysis

To validate the results of HRM analysis, the HRM-PCR products were purified using Multifunction DNA Purification Kit (Bioteke, China). And then the purified PCR products were sequenced in both directions with the same PCR primers on an ABI 3730 DNA Sequencer (Applied Biosystems). The CodonCode Aligner 4.0.4 (CodonCode Co., USA) was used to proofread, assemble the contigs, and generate consensus sequences. Complete ITS2 sequences were retrieved according to hidden Markov model based model analysis. The sequence alignment was performed with Clustal W. The tree-based method was used for species identification analyses: the neighbor-joining (NJ) tree was conducted by MEGA 5.0 with 1000 bootstrap replicates, and the bootstrap value above 50% was shown. All the sequences were also submitted to DNA barcoding system for identifying herbal medicine (http://www.tcmbarcode.cn/en/) to verify the HRM results.

## Additional Information

**How to cite this article**: Song, M. *et al*. Applying high-resolution melting (HRM) technology to identify five commonly used *Artemisia* species. *Sci. Rep.* **6**, 34133; doi: 10.1038/srep34133 (2016).

## Figures and Tables

**Table 1:** Plant materials used in this study.

Species	Origin	Locality	Voucher No.	Genbank No.
*Artemisia argyi*	Original plant	Guangxi China	SM001MS01~03	KX185157~59
*A. argyi*	Commercial product	Anhui, Sichuan, Hebei, Hubei China	SM001MS04~16	KX185160~72
*Artemisia annua*	Original plant	Chongqing, Guangxi, Beijing China	SM002MS01~03	KX219663~65
*A. annua*	Commercial product	Beijing, Hubei China	SM002MS04~19	KX219666~81
*Artemisia lavandulaefolia*	Original plant	Jiangxi China	SM003MS01 SM003MS02	KX219705 KX219706
*A. lavandulaefolia*	Commercial product	Neimenggu, Beijing, Anhui, Guangxi China	SM003MS03~13	KX219707~17
*Artemisia indica*	Original plant	Jiangsu China	SM004MS01~03	KX219695~97
*A. indica*	Commercial product	Henan, Hubei, Anhui China	SM004MS04~10	KX219698~704
*Artemisia atrovirens*	Original plant	Sichuan China	SM005MS01 SM005MS02	KX219682 KX219683
*A. atrovirens*	Commercial product‘	Anhui, Hubei, Henan, Guangxi China	SM005MS03~13	KX219684~94
